# Profiling of Tumor-Infiltrating Immune Cells and Their Impact on Survival in Glioblastoma Patients Undergoing Immunotherapy with Dendritic Cells

**DOI:** 10.3390/ijms25105275

**Published:** 2024-05-12

**Authors:** Nataly Peres, Guilherme A. Lepski, Carla S. Fogolin, Gabriela C. M. Evangelista, Elizabeth A. Flatow, Jaqueline V. de Oliveira, Mariana P. Pinho, Patricia C. Bergami-Santos, José A. M. Barbuto

**Affiliations:** 1 Department of Psychiatry, Medical School, Universidade de Sao Paulo, Sao Paulo 05403-010, Brazil; natalyperes@usp.br; 2 LIM 26, Hospital das Clínicas HCFMUSP, Faculdade de Medicina, Universidade de Sao Paulo, Sao Paulo 05403-000, Brazil; 3 Department of Neurosurgery, Eberhard-Karls University, 72074 Tuebingen, Germany; 4 Department of Immunology, Instituto de Ciencias Biomedicas, Universidade de Sao Paulo, Sao Paulo 05508-000, Brazil; carlafogolin@gmail.com (C.S.F.); gabrielacoeli@usp.br (G.C.M.E.); eaflatow@gmail.com (E.A.F.); jaquelinevazdeoliveira@gmail.com (J.V.d.O.); marianappinho@gmail.com (M.P.P.); patriciabergami@yahoo.com.br (P.C.B.-S.); jbarbuto@icb.usp.br (J.A.M.B.); 5 Laboratory of Medical Investigation in Pathogenesis and Targeted Therapy in Onco-Immuno-Hematology (LIM-31), Department of Hematology, Hospital das Clínicas HCFMUSP, Faculdade de Medicina, Universidade de Sao Paulo, Sao Paulo 05403-000, Brazil

**Keywords:** glioblastoma, tumor infiltrate, tumor microenvironment, PD-L1, CD86, immunotherapy, patient survival, dendritic cells

## Abstract

Glioblastomas (GBM) are the most common primary malignant brain tumors, comprising 2% of all cancers in adults. Their location and cellular and molecular heterogeneity, along with their highly infiltrative nature, make their treatment challenging. Recently, our research group reported promising results from a prospective phase II clinical trial involving allogeneic vaccination with dendritic cells (DCs). To date, six out of the thirty-seven reported cases remain alive without tumor recurrence. In this study, we focused on the characterization of infiltrating immune cells observed at the time of surgical resection. An analytical model employing a neural network-based predictive algorithm was used to ascertain the potential prognostic implications of immunological variables on patients’ overall survival. Counterintuitively, immune phenotyping of tumor-associated macrophages (TAMs) has revealed the extracellular marker PD-L1 to be a positive predictor of overall survival. In contrast, the elevated expression of CD86 within this cellular subset emerged as a negative prognostic indicator. Fundamentally, the neural network algorithm outlined here allows a prediction of the responsiveness of patients undergoing dendritic cell vaccination in terms of overall survival based on clinical parameters and the profile of infiltrated TAMs observed at the time of tumor excision.

## 1. Introduction

Glioblastoma (GBM), recognized as one of the most aggressive forms of brain cancer, accounts for approximately 47% of all primary brain tumors [1]. Despite advances in medical science, glioblastoma patients face grim prospects, with median survival rates hovering around 14 to 16 months post-diagnosis [2]. The likelihood of recurrence is exceedingly high, with over 90% of patients experiencing tumor regrowth, often within months [3]. This poor outcome is partly attributed to glioblastoma’s notorious cellular heterogeneity, its capacity for rapid proliferation, and the unique challenges posed by the central nervous system [4].

Currently, the standard treatment protocol for glioblastoma involves a multimodal approach that combines maximal safe surgical resection, followed by concurrent radiotherapy and chemotherapy, typically with Temozolomide [5]. Despite this aggressive treatment regimen, the inflexibly high mortality rate associated with glioblastoma underscores the urgent need for innovative therapeutic strategies and highlights the critical importance of ongoing research into more effective treatments.

The integration of immunotherapy, particularly through dendritic cell (DC) vaccine strategies [6], into the treatment landscape for glioblastoma has opened new avenues for combating this aggressive type of cancer [7]. Centered around the pivotal role of the tumor microenvironment and the complex interplay of immune cell infiltration, these innovative approaches seek to harness the body’s immune system to recognize and attack glioblastoma cells [8,9]. As clinical trials continue to explore the efficacy of dendritic cell vaccines, there is growing optimism about their potential to improve patient outcomes, minimize recurrence, and pave the way for personalized therapies that exploit the unique immunological landscape of each patient’s tumor [10]. Recently, our research group reported promising results from a prospective phase II clinical trial involving allogeneic vaccination with dendritic cells. We reported prolonged overall survival in astrocytoma grade 4 (20 to 60 months, hazard ratio 0.18), as well as in glioblastoma (16 to 28 months, hazard ratio 0.5). To date, six out of the thirty-seven reported cases remain alive without tumor recurrence [11].

Nonetheless, an important component of the tumor microenvironment of GBM cannot be overstated. Tumor-associated macrophages (TAMs) are the most abundant immune cell population in the context of glioblastoma, constituting up to 40% of the tumor volume [12]. TAMs can play a crucial role in the success or failure of immunotherapies, particularly when considering the development and application of dendritic cell vaccine strategies [13,14]. These macrophages (MΦ), often persuaded by tumor cells, can shift the immune landscape towards one that favors tumor persistence and immune evasion [15]. Their role in modulating the immune response is a double-edged sword that can either enhance or inhibit the effectiveness of immunotherapeutic interventions [16].

Although they are classically considered critical effector cells during immune defense, several studies have demonstrated a clear role for tumor-associated macrophages in supporting multiple aspects of tumor progression [17,18]. A possible explanation for these disparate activities during normal tissue homeostasis and tumorigenesis lies in their plasticity, which is necessary for their roles in the physiology of the inflammatory reaction [19]. These cells have their functional state clearly modified by the physiological conditions of the tissue where they are found. Considering the extremes of this functional modulation, macrophages (MΦ) have been described as MΦ-M1, classically activated, or MΦ-M2, alternatively activated [20]. However, it is critical to note that this classification may be an oversimplification of their biology and is often used for didactic purposes, being observed when macrophages are differentiated in vitro under well-defined conditions [21,22]. Nonetheless, the plasticity of macrophages in vivo is much more complex and is adapted to meet the needs of the tissues and can assume different characteristics within a spectrum that goes from activation to regulation.

In the context of dendritic cell vaccines, the presence and polarization of TAMs can critically determine the robustness of the elicited anti-tumor T-cell response [23]. Therefore, characterizing TAMs within the glioblastoma microenvironment is pertinent for tailoring vaccine strategies that can navigate and potentially reverse the immunosuppressive milieu they create. Through such understanding, it is possible to identify specific targets and develop strategies that modify TAM behavior, supporting a more conducive environment for immunotherapies to achieve their full potential in treating this difficult cancer.

Thus, the present study aimed to characterize the phenotype of macrophages (CD45+HLA-DR+CD11b+CD14+) recovered from tumor samples from patients diagnosed with GBM and submitted to immunotherapy with dendritic cells, correlating their frequency and phenotype with the survival of these patients.

## 2. Results

Following mechanical disruption and enzymatic digestion, GBM tissues were subjected to immunostaining using antibodies targeted for hematopoietic (CD45), myeloid (CD11b and CD14), and T lymphocyte (CD3) markers, in addition to HLA-DR antibodies. Flow cytometry was utilized to delineate the cellular composition of suspensions of single cells derived from 21 patient samples in order to specifically identify the prevalence of TAMs (CD45+CD11b+HLA-DR+CD14+). Additionally, the presence of T lymphocytes (CD45+CD3+) was assessed in 12 of these samples.

**Figure 1:**
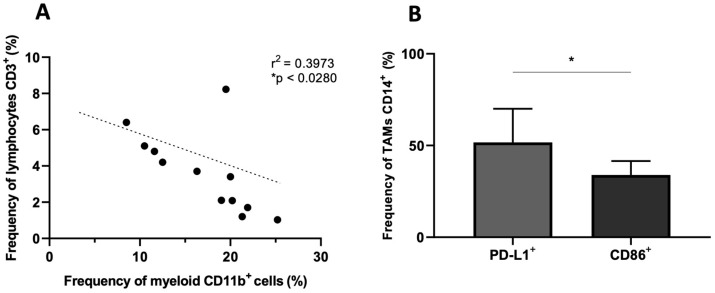
Frequency of tumor-associated immune cells and the activation status of TAMs. (**A**) Correlation between the frequency of CD45+CD3+ and CD45+CD11b+ GBM-infiltrating immune cells (N = 12; * *p* < 0.05). (**B**) Frequency of TAM (CD45+HLA-DR+CD11b+CD14+) positive for PD-L1 and CD86 markers. (N = 21; * *p* < 0.05).

Initially, the analysis sought to unravel potential correlations between lymphoid and myeloid cell populations within the tumor. Figure 1A reveals a notable negative correlation between the frequency of tumor-infiltrating CD45+CD11b+ cells and CD45+CD3+ T cells. Subsequently, the analysis focused on TAMs, particularly looking at the expression levels of CD86 and PD-L1, both markers indicative of their activation state and potential to stimulate lymphocytes. Figure 1B reveals a significantly greater frequency of TAMs positive for PD-L1 in comparison to the CD86 marker.

In order to investigate the existence of a correlation between the immunological data and the survival of patients included in this study, different strategies were employed. Firstly, a non-parametric method was employed to explore correlations. The data are presented in Supplementary Material Table S1, which shows Spearman’s coefficient values restricted to comparisons concerning survival data to enhance clarity. A coefficient is considered significant if it is greater than 0.6. Accompanying these coefficients are the corresponding *p*-values. Significance levels were visually distinguished: red denotes *p* < 0.05, indicating statistical significance, while orange denotes a higher level of statistical significance, marked by *p* < 0.01 or *p* < 0.001. The analysis revealed statistically significant correlations for survival post-tumor recurrence with the variables CD45+PD-L1+ (leukocyte cells positive for PD-L1) and CD45+CD11b+CD14+ PD-L1+ (TAMs positive for PD-L1). However, such significance was not observed in relation to the variable CD86+.

**Figure 2:**
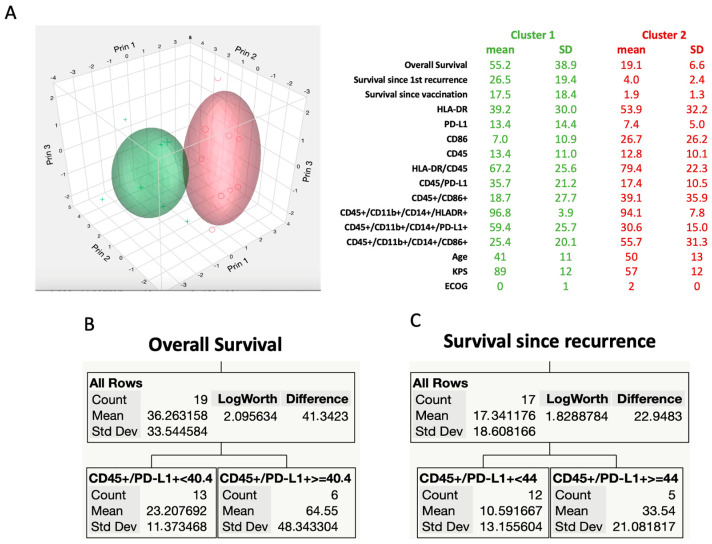
K-means cluster and decision tree analysis. (**A**) K-means cluster. K was defined as 2 in order to obtain one group (cluster) with long overall survival (cluster 1, in green) and another with short overall survival (cluster 2, in red). The analysis reports the mean and standard deviations of each variable studied in each of the two identified groups (summarized in the table alongside). (**B**) Decision tree. Partition of overall survival data into 1 level based on the expression of CD45+/PD-L1+ being less than or greater or equal to 40.4%. The analysis refers to the mean of overall survival, standard deviations, and a *p*-value in terms of LogWorth of the difference between the groups. (**C**) Similarly, the same type of analysis for survival data since the 1st recurrence coincidentally resulted in 1 level of partition and was based on the expression of CD45+/PD-L1+ but with a slightly different cutoff value (44%), which also reached statistical significance (LogWorth *p*-value of 1.82).

Next, an attempt was made using the “k-means clustering” analysis (Figure 2A) that allowed grouping into k groups, estimating the classification of the data as belonging to each of the groups based on Euclidean distance from centroids in each group or cell (Voronoi Cell). In this case, k was defined as 2, with the intention of having one group with long overall survival and another with short overall survival. The analysis reports the mean and standard deviations of each variable studied in each of the two identified groups (summarized in the table alongside). The graph thus shows the dispersion around two clusters, where each axis represents the first three principal components (“prin”). The size of the ellipse is proportional to the counts within the clusters, and the volume represents the density of 50% of the data around the centroids. It is noted that cluster 1 (green) has much more favorable overall survival, survival since tumor recurrence, and survival since the start of vaccination than cluster 2 (red). Interestingly, the more favorable group includes patients who are younger, have a higher KPS and lower ECOG, and, in immunological terms, have leukocytes (CD45+) and macrophages (CD45+CD11b+CD14+) with higher expressions of PD-L1 and lower expressions of CD86.

Following this, we used the decision tree method, a non-parametric supervised learning machine technique used for classification and regression. The data were recursively divided by the relationship between predictors (clinical and immunological variables) and response (survival data). This data mining technique is usually used to explore relationships in the absence of a good baseline model. The partition criterion (segregation among the data) is based on the LogWorth statistic, that is, −log10 (*p*-value). Continuous variables are considered for partitioning in terms of an optimal cutoff value calculated by the receiver operating characteristic curve (ROC curve). Among the various partitions and different levels, the analysis reports the best possible partition. In terms of overall survival (2B), the sample was divided between those with CD45+PD-L1+ less than 40.4%, who lived for an average of 23 months (SD 11.4 months), and those with CD45+PD-L1+ greater than or equal to 40.4%, who lived for 64.6 ± 48.3 months (LogWorth 2.09—equivalent to a *p* = 0.00812). Similarly, in the analysis of survival since tumor recurrence, reported in Figure 2C, the sample also coincidentally divided according to the expression of CD45+PD-L1+, dividing between those with positivity less than 44%, who survived for 10.6 ± 13.2 months, and those with CD45+PD-L1+ greater than or equal to 44%, whose survival was 33.5 ± 21.1 months (LogWorth 1.8288—equivalent to a *p* = 0.01483). For the partition for survival since the start of vaccination, the analysis did not result in statistically significant values.

**Figure 3:**
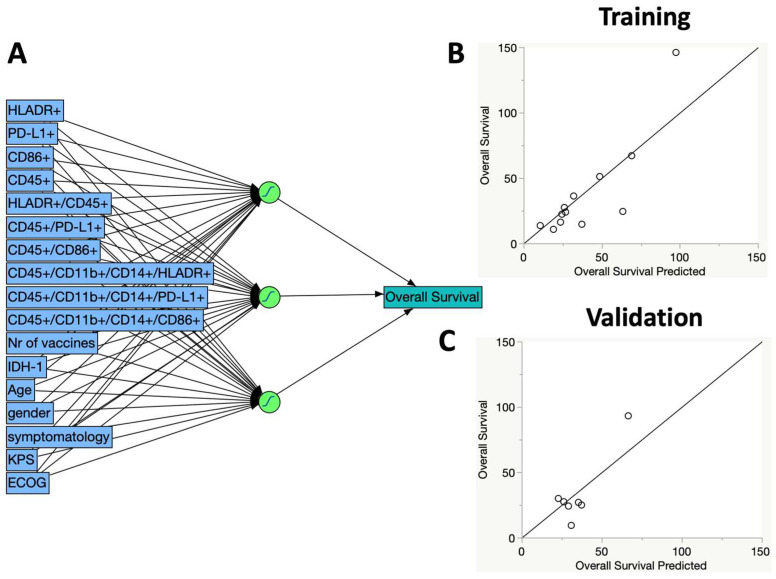
Survival prediction analysis using neural networks. In Panel (**A**), the diagram illustrates the analysis with added variables to the model in blue on the left, activation functions in green, and the final variable, overall survival, on the right. The algorithm randomly splits the original data for training and validating the model. Panels (**B**,**C**) show graphs comparing the actual overall survival times with the values calculated (predicted) by the training phase (**B**) and validation phase (**C**).

Aiming to propose a predictive model of survival, we employed another machine learning algorithm, called the artificial neural network. Validation of the algorithm was tested according to the random holdback method, featuring three hidden nodes (non-linear functions), a 0.33 holdback proportion, one layer, and five validations (random seeds). In Figure 3A, the diagram of the analysis shows the variables added to the model (in blue on the left), the activation functions (symbols in green), and the final variable, overall survival, on the right. The algorithm randomly divides the original data for training and model validation. In Figure 3B,C, the chart with the actual values of overall survival and those calculated (predicted) are seen, both in the training phase of the algorithm (B) or in its validation (C). The summary statistics of the model showed similar R square values for both the training phase and the validation phase, denoting good prediction reproducibility. It is crucial to highlight that simulations adding or removing variables from the model resulted in lower R square values.

**Figure 4:**
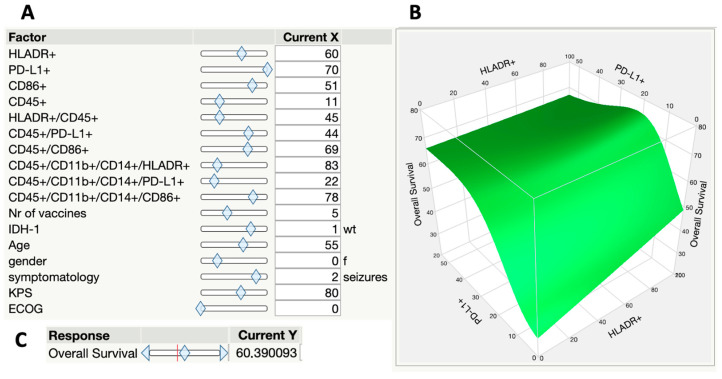
Application of the neural network prediction model. In (**A**), the input of new values for each of the variables of interest, whether they are continuous numerical variables (such as age, percentage of cells positive for HLA-DR, etc.), ordinal variables (KPS, ECOG), or even categorical variables (such as sex and symptomatology). In (**B**), the reconstructed surface plot with 3 of the variables of interest. In this case, overall survival was kept fixed on the *Y*-axis, PD-L1+ on the *x*-axis, and HLADR+ on the *z*-axis. In (**C**), the result of the estimated overall survival (“Current Y”, in this case, 60.4 months), with an accuracy of 71%, corresponds to the R-square of the model.

The application of this model allows for the input of new values for the initial variables (indicated on the right side in Figure 4), including both continuous numerical variables (such as age, percentage of cells positive for markers of interest, etc.) and ordinal variables (KPS, ECOG), as well as categorical variables (such as sex and symptoms). The surface graph (on the left) can be reconstructed with three of the variables of interest. In this case, overall survival was set fixed on the *Y*-axis as the final variable of interest. After entering the new values for each variable, the algorithm calculates the overall survival (“Current Y”, in months), with an accuracy of 71% (which corresponds to the model’s R square).

## 3. Discussion

In this study, we analyzed the presence of immune system cells within the glioblastoma tumor microenvironment. In the 21 patient samples examined, a wide variation in the frequency of hematopoietic (CD45+) cells was observed. This variability was expected, as such infiltration is governed by numerous sensitive elements of the tumor microenvironment, which is uniquely shaped in each individual [4,24]. Among the hematopoietic (CD45+) cells, myeloid-derived cells (CD45+CD11b+), which include neutrophils, certain dendritic cell subgroups, monocytes, macrophages, and microglia, prevailed over T lymphocytes (CD45+CD3+). This higher frequency of CD11b+ cells is not surprising, as the literature data point to this characteristic in GBM [25]. However, a direct negative correlation between myeloid cells and T lymphocytes observed here is not known to be described in the literature.

In various tumor types, the presence of certain myeloid cells and the low frequency of T lymphocytes have been linked to the tumor’s immunosuppressive environment [26]. It is pertinent to note that, although the literature emphasizes the immunosuppressive role of TAMs [16], tumor-associated macrophages can have quite diverse phenotypes and functions, and in some cases, they may play an anti-tumoral role [27]. It is worth noting the same functional ambivalence can be applied to T lymphocytes, which, in addition to anti-tumoral roles, can act as immunosuppressors, particularly when exhibiting a regulatory T lymphocyte phenotype, impeding effective anti-tumoral responses [28].

Our data corroborate that, in GBM, TAMs constitute the main immune cell type within the tumor microenvironment. This leads us to consider, given the cellular plasticity of this group, whether these macrophages might be acting as supporters of an exclusively pro-tumoral response, as often associated, or as contributors to anti-tumoral immunity at some level as well.

Pan and colleagues discuss the dual nature of TAMs in a study published in 2020, showing that tumor-associated macrophages can exhibit both tumor-promoting and inhibitory properties within the tumor microenvironment, depending on their polarization state [29]. TAMs polarized to an M1-like profile may exhibit anti-tumor functions, potentially leading to better outcomes for patients, while TAMs polarized to an M2-like profile are typically associated with immunosuppression and tumor promotion. It is highlighted that the balance between these two states can be influenced by the tumor microenvironment and therapeutic interventions, with the possibility of reversing both states.

Regarding the duality of TAMs in different tumors, an interesting study conducted with patients diagnosed with triple-negative breast cancer (TNBC) found that TAMs displayed high levels of CD163+ and, therefore, an M2-like phenotype. It was observed that the expression of CD163+ in the tumor stroma was associated with the absence of hormone receptors and an increase in the aggressive characteristics of this type of breast cancer [30]. However, recent studies have also indicated that soluble factors obtained from TNBC cell lines could modulate monocytes into a mixed population of M1-like and M2-like macrophages simultaneously. Unlike the classic M2-polarized macrophage, these TAMs had upregulated genes associated with the M1 profile and secreted pro-inflammatory cytokines, such as IL-6 [31].

Collectively, these data suggest a complex interaction between macrophages and the cells of the tumor microenvironment. Therefore, the direct cytotoxic activity of macrophages, often underestimated in focus given to T cell cytotoxicity, deserves to be more broadly studied. In this sense, some studies conducted both in vitro and in vivo reported that the proper stimulation of macrophages can result in tumor regression. For example, in a small cohort of patients with pancreatic ductal adenocarcinoma, tumor regression correlated with TAM infiltration following treatment with chemotherapy and a CD40 agonist [32]. Co-culture experiments in vitro revealed the direct elimination of tumor cells by TAMs themselves [33]. These studies underline that tumor-associated macrophages should be considered effector cells in their own right when properly activated. This implication is supported by results from an animal model in which CSF-1R is blocked, thereby affecting their functional polarization and blocking their glioma progression [34].

Thus, to better understand the quality of TAMs present in the tumor infiltrate of patients diagnosed with GBM, we investigated the phenotype of these macrophages. Our data showed that the TAM phenotype varied among patients, but generally, these cells exhibited a high expression of PD-L1 and a low expression of the CD86 marker.

In terms of survival, we observed a positive correlation between patient survival and the expression of PD-L1 by immune cells present in the tumor microenvironment. The physiological expression of PD-L1, particularly in immune system cells, is a crucial aspect of immune response regulation. Under normal conditions, PD-L1 expression serves to maintain immunological homeostasis, prevent autoimmunity, and facilitate immunological tolerance; for example, during pregnancy or in the presence of healthy tissues and transplanted organs [35].

One of the main pathways of the induction of PD-L1 expression is through activation by inflammatory cytokines. IFN-γ, produced by activated T cells, is a potent inducer of PD-L1 expression. This regulation represents a negative feedback mechanism, whereby upon recognizing and responding to a pathogen, T cells activate the production of IFN-γ, which, in turn, induces the expression of PD-L1 in target cells, including immune and tumor cells. PD-L1 expression then acts to “brake” the immune response, potentially limiting tissue destruction and preventing excessive autoimmune responses [36]. In addition to cytokine induction, PD-L1 expression can also be increased in immune system cells, such as macrophages and dendritic cells, when they are activated. For example, the activation of antigen-presenting cells through the recognition of pathogen-associated molecular patterns (PAMPs) by means of PRRs can lead to PD-L1 expression, contributing to regulating the amplitude of the immune response initiated by these cells [37].

Thus, we can infer that a more activated tumor microenvironment naturally expresses more PD-L1. The kinetics of PD-L1 expression in tumors are complex and can vary considerably among different types of cancer, as well as among patients with the same type of cancer. The variability in PD-L1 expression can be influenced by various factors, such as (i) the genetics of the tumor, where some mutations in signaling pathways, such as the PI3K/AKT pathway, can result in increased PD-L1 expression [38]; (ii) the tumor microenvironment, where inflammatory or anti-inflammatory cytokines, such as IFN-γ and IL-10, are potent inducers of PD-L1 expression in cells [39]; and (iii) the interaction of neoplastic cells with the immune system [40].

A hypothesis that can be formulated is that, in tumors located outside the CNS, the immune system cells can probably infiltrate more quickly, and the physiology of PD-L1 may then correspond to this “brake” on the inflammatory immune response [41]. However, in GBM, due to particular conditions and relative isolation of the CNS, perhaps the kinetics of PD-L1 is different, such that the immune system cells can only infiltrate the tumor in later phases when there is a rupture or facilitation of the blood–brain barrier. In this way, the PD-L1 found in the infiltrating immune cells of GBM observed in this study may be highly expressed, indicating widespread cellular activation and not necessarily representing a “brake” on the anti-tumor response.

Another possible conjecture could be that the high expression of PD-L1 in tumor-associated macrophages (TAMs) and its correlation with improved survival in these patients might indeed reflect a more immunosuppressive tumor microenvironment, a status that might have been changed under influence of the cellular immunotherapy. In tumors that exhibit high PD-L1 expression, there is often an increased presence of molecules associated with immune checkpoints, reflecting an advanced strategy for immune evasion and suppression. Although this might seem detrimental to anti-tumor immunity, it also indicates that the tumor microenvironment is intrinsically more immunogenic and, therefore, might be more susceptible to interventions aimed at reversing this immunological suppression [42].

This scenario aligns with observations in patients harboring lung cancer who respond very well to immunotherapy with PD-1 inhibitors, which prevent the ligand from binding to its receptor. This strategy has proven particularly effective in certain types of non-small-cell lung cancer, where it significantly improved survival rates compared to conventional therapies [43]. However, this pattern does not replicate in GBM, where immunotherapy with immune checkpoint inhibitors fails to achieve notable outcomes in tumor reduction or in prolonging patient survival [44]. This phenomenon is highlighted in a phase III clinical trial within a cohort of 369 patients with recurrent glioblastoma, where immunotherapeutics like Nivolumab—an IgG4 monoclonal antibody that blocks the PD-1 receptor—did not prove to be superior to other treatments, such as Bevacizumab (an inhibitor of the VEGF angiogenic factor) [45].

It is crucial to consider the implications of dendritic cell additional activation highlighted by recent studies when evaluating immunotherapeutic approaches. Our study suggests that modulating the activation state of tumor-associated macrophages can significantly enhance the efficacy of dendritic cell-based immunotherapy.

To illustrate this point, a study on Imiquimod, a known activator of Toll-like receptor 7 (TLR7), demonstrated its critical role in enhancing dendritic cell function for genetic immunization against HIV-1 p55 Gag [46]. Imiquimod’s ability to stimulate DCs is instrumental for effective antigen presentation and T-cell activation. By integrating novel adjuvants, such as TLR agonists, similar to Imiquimod, it is possible to amplify the immune response, thus enhancing DC vaccine effectiveness. However, the success of DC vaccines, especially in treating complex conditions, like glioblastoma, must effectively navigate and counteract the immunosuppressive tumor microenvironment.

It is also important to note that the activation of dendritic cells and TAMs needs careful management and a more stringent definition. Our findings indicate that while certain markers, such as PD-L1, correlate with better outcomes, others, such as CD86, could be associated with worse outcomes, results that could be interpreted as counterintuitive since CD86 is usually used as a marker of DC “activation” and PD-L1 as one of “suppressive” activity. Therefore, the selective targeting of pathways to optimize the activation and function of these immune cells is critical for improving the therapeutic success of dendritic cell vaccines in glioblastoma patients. This nuanced approach highlights the complexity of the immune microenvironment in glioblastoma and underscores the need for strategies that can effectively harness the potential of immune cells to combat this aggressive cancer.

Recently, several studies have sought to investigate the prognostic value of PD-L1 expression in cancer patients. However, the results remain inconclusive. A meta-analysis by Yang et al. [47] indicates that the matter remains contentious regarding melanoma, for example. Their findings suggest that PD-L1 expression does not predict a worse prognosis in melanoma patients, while high PD-L1 expression was also linked to the absence of lymph node metastasis in these patients. In contrast, in patients with adrenocortical carcinoma, no correlation has been found between PD-L1 expression and survival [48].

For glioma, data are limited and also controversial. Some studies suggest that high PD-L1 expression in glioma cells correlates with poor prognosis. Zhu et al. [49], for instance, analyzing gene expression data from patients diagnosed with glioma from databases, like The Cancer Genome Atlas (TCGA) and the Chinese Glioma Genome Atlas (CGGA), found a positive correlation between tumor PD-L1 expression and the infiltration of alternatively activated (M2) macrophages, along with an unfavorable prognosis. A 2013 study by Liu et al. [50] found that the upregulation of PD-L1 by neurons in the brain tissue adjacent to the tumor is positively associated with survival in GBM, while the lack of neuronal PD-L1 expression was linked to a high PD-L1 expression in tumor cells and an unfavorable prognosis.

Beyond the positive correlation found between the elevated PD-L1 expression in recovered TAMs and increased survival of GBM patients in this study, it is necessary to observe the curious negative correlation found between low patient survival and a high expression of the CD86 marker. Some studies suggest that the presence of pro-inflammatory (M1) phenotype macrophages, which can express CD86 in certain contexts, is linked to a more favorable prognosis [51,52]. CD86 is a co-stimulatory molecule expressed by activated macrophages and other antigen-presenting cells. It is known that the expression of this molecule is especially induced in response to pathogens or inflammatory signals, playing a crucial role in the activation and differentiation of T cells. Therefore, in the context of cancer, the binding of CD86 to CD28 on T lymphocytes can assist in their activation, enhancing an effective anti-tumor immune response. However, the interaction of CD86 with CTLA-4, a regulatory receptor also present in some T cells, can suppress the activation of these effector cells, contributing to immunosuppression and allowing the tumor to evade immune surveillance [53,54].

Thus, the expression of CD86 on APCs can contribute both to the promotion and the inhibition of tumor growth [55,56]. In our study, we found a negative correlation between survival and CD86 expression. We hypothesize that this fact is due to the competitive binding of CD86 to CTLA-4, which occurs at a higher affinity than that for CD28, thus prevailing an inhibitory immune response. Thus, the presence and role of CD86 in the tumor microenvironment, as well as the PD-L1 marker, prove to be complex and can vary, depending on the type of cancer and the immunological status of the tumor [57].

In order to understand the correlations between clinical and immunological data, various strategies were employed. 

First, the analysis of “k-means clustering”, which partitioned patients into two groups based on survival (favorable and unfavorable), revealed that the group with higher survival exhibited greater infiltration of TAM (CD45+CD11b+CD14+) and expressed high levels of PD-L1 and low levels of CD86.

Second, in the partition analysis by decision tree, the algorithm selects, among the measured variables, those with the greatest weight in the composition of the final outcome (survival), and the data indicated that the expression level of CD45 and PD-L1 markers was the most important factor in determining overall survival, with a value greater than the cutoff of 40.4% implying significantly longer survival, as opposed to the group with an expression lower than 40.4% (65 vs. 23 months). A similar partition based on PD-L1 expression was found when analyzing survival since the first recurrence, although the cutoff value was slightly different (44%, 10 vs. 33 months for lower or higher than the cutoff value, respectively). In this analysis, the most decisive influence on survival was the expression of PD-L1 in general hematopoietic cells, rather than specifically in TAM.

Finally, we applied a more complex model based on artificial neural networks [58]. This modeling is more accurate as it considers multiple factors in concurrent or serial non-linear functions. Indeed, modeling by neural networks has recently allowed the interpretation and prediction of extremely complex natural phenomena, ranging from climate modeling to language decoding [59]. Our model was able to predict 71% of survival, with acceptable reproducibility. It is interesting to note that considering each of the independent variables individually or in groups (as in linear models) did not result in a reliable prediction of survival. It was only with the consideration of the entire constellation of clinical and immunological factors that the set of variables began to make sense, illustrating the complex nature of these interactions. Finally, our analysis reinforces the emerging view that tumor-associated macrophages (TAMs) are not exclusively promoters of immunosuppression and tumor growth [60]. In contrast to the prevalent notion of TAMs as pro-tumoral cells, our data point to a greater complexity, where TAMs can exhibit a range of functional states, including those in which the cells perform anti-tumor activities and are associated with a more favorable prognosis in GBM patients. This understanding challenges the binary M1/M2 concept and underscores the need for therapeutic approaches that consider the functional versatility of TAMs, aiming to maximize clinical outcomes in cancer treatments.

## 4. Materials and Methods

### 4.1. Patient Recruitment and Ethics

Patients in our phase I/II prospective trial on allogenic DC vaccination for GBM at our institution were recruited as described previously [11]. All procedures were approved by the institutional Ethics Committee and the National Research Council at the University of São Paulo (approval No. 58882116.7.3001.0065), and patients were enrolled after providing written informed consent. Clinical and laboratory data were collected prospectively and anonymously and recorded using the RedCap platform hosted at Hospital das Clínicas, Medical School, University of São Paulo (https://redcap.hc.fm.usp.br, accessed on 5 December 2023) [61,62].

### 4.2. Tumor Sample Processing

The fresh tumor sample obtained from surgical resection was minced and digested with collagenase type VIII (0.56 mg/mL; Sigma-Aldrich, San Luis, MI, USA) under agitation at 37 °C for 2 h. Cell suspensions were separated from the non-digested fragments using sterile gauze and washed twice in RPMI-1640. Subsequently, they were frozen at −80 °C in an appropriate medium for later immunophenotyping analysis. In parallel, the tumor supernatant (SNTum) was also reserved for further use in in vitro assays.

### 4.3. Immunophenotyping of Tumor Cell Infiltrate by Flow Cytometry

The tumor suspensions were thawed, washed in 1× phosphate-buffered saline (PBS), and centrifuged at 1200 rpm and 18 °C for 10 min. Next, they were incubated for 30 min at 4 °C with specific fluorescent antibodies (CD45, HLA-DR, CD11b, CD14) or isotype controls (BD Biosciences, San Jose, CA, USA) to determine the frequency of macrophages and the expression of PD-L1 and CD86 molecules (associated, respectively, with inhibition and stimulation of the T lymphocyte response) in these cells. After staining, the cells were washed and resuspended in 200 µL of 1× PBS in order to be acquired on the FACSCanto II flow cytometer using the FACS Diva software v.8.0.1 (BD Biosciences, Franklin Lakes, NJ, USA). The data obtained were subsequently analyzed in FlowJo software v.10.10 (Tree Stars, Ashland, OR, USA).

### 4.4. Survival Analysis

We conducted a multivariate analysis correlating immunological variables and survival data. Next, we applied the “k-means clustering” method [63], estimating the classification of the data belonging to each group based on the Euclidean distance from the centroids of each group or cell (Voronoi Cells). In this case, we defined k as 2, aiming to have one group with long overall survival and another with short overall survival. The analysis reports the means and standard deviations of each variable studied in each of the two identified groups. We also analyzed survival using a hierarchical artificial intelligence algorithm called a decision tree. This is a data mining technique used to explore relationships in the absence of a good baseline model. The partition criterion is based on the LogWorth statistic, that is, −log10 (*p*-value). Continuous variables are considered for partition in terms of an optimal cutoff value calculated by the ROC (receiver operating characteristic curve). Among the various possible partitions and different levels, the analysis reports the best possible partition. Subsequently, we applied another machine learning method called artificial neural networks [62]. This model aims to predict an outcome (in this case, overall survival) based on a wide database analyzed in stages (layers) and at each layer analyzed by different non-linear models, called “nodes” or “neurons”, similar to a neuron in a biological network. A fraction of the data (holdback proportion) is used for model validation. In this case, we used a fraction of 0.33, and the best model after several tests resulted in 3 nodes in 1 layer, with 5 validations (random seeds). As in other analyses, this model reports an “R square”, which represents the model’s prediction probability, where values closer to 1 indicate better modeling; similar R-square values in the training and validation phases denote good prediction reproducibility.

## 5. Conclusions

Up to this point, attempts to correlate survival in malignant gliomas with genetic and biomolecular aspects of the tumor have only made very limited advances, with the exception of IDH-1. Our study highlights the crucial role of the immune system and its interactions within the tumor’s microenvironment in affecting survival outcomes. We report significant findings on the beneficial impact of PD-L1 expression in tumor-infiltrating macrophages and the detrimental effects of CD86-positive macrophages on overall survival among patients treated with dendritic cell-based immunotherapy. Furthermore, we developed a sophisticated neural network model that predicts survival based on the analysis of the tumor-infiltrating immune population. This model achieved an acceptable success rate (71%) and high reproducibility. The role of the immune system in the survival of cancer patients has been underemphasized so far, and the development of targeted immunotherapies could be greatly accelerated if we better understand these mechanisms for both prognostic and therapeutic purposes.

## Data Availability

All clinical and laboratory data were collected according to the national ethical rules and legislation and are available for analysis upon request.

## Supplementary Materials

The following supporting information can be downloaded at: https://www.mdpi.com/article/10.3390/ijms25105275/s1.
