# Supplementary material for: Profiling of Tumor-Infiltrating Immune Cells and Their Impact on Survival in Glioblastoma Patients Undergoing Immunotherapy with Dendritic Cells
Source: Int J Mol Sci. 2024 May 12;25(10):5275. doi: 10.3390/ijms25105275 (PMC11121326; doi:10.3390/ijms25105275)
Supplement: Supplementary file 1 [file ijms-25-05275-s001.zip › ijms-2997449-supplementary.pdf]

| Variable | by Variable | Spearman ρ | Prob>\|ρ\| |
|---|---|---|---|
| Overall Survival | living cells | -0,1264 | 0,6062 |
| Overall Survival | HLADR+ | -0,0614 | 0,8027 |
| Overall Survival | PD-L1+ | 0,0931 | 0,7047 |
| Overall Survival | CD86+ | -0,2264 | 0,3513 |
| Overall Survival | CD45+ | -0,2726 | 0,2588 |
| Overall Survival | HLADR+/CD45+ | -0,0290 | 0,9063 |
| Overall Survival | CD45+/PD-L1+ | 0,3098 | 0,1968 |
| Overall Survival | CD45+/CD86+ | -0,0237 | 0,9233 |
| Overall Survival | CD45+/CD11b+ | -0,1659 | 0,4974 |
| Overall Survival | CD45+/CD11b+/CD14+ | -0,1790 | 0,4634 |
| Overall Survival | CD45+/CD11b+/CD14+/HLADR+ | 0,0707 | 0,7735 |
| Overall Survival | CD45+/CD11b+/CD14+/PD-L1+ | 0,3721 | 0,1167 |
| Overall Survival | CD45+/CD11b+/CD14+/CD86+ | -0,2361 | 0,3306 |
| Survival since 1st recurrence | living cells | -0,1936 | 0,4565 |
| Survival since 1st recurrence | HLADR+ | 0,2181 | 0,4003 |
| Survival since 1st recurrence | PD-L1+ | 0,4510 | 0,0692 |
| Survival since 1st recurrence | CD86+ | -0,1569 | 0,5477 |
| Survival since 1st recurrence | CD45+ | 0,0270 | 0,9182 |
| Survival since 1st recurrence | HLADR+/CD45+ | 0,1961 | 0,4507 |
| Survival since 1st recurrence | CD45+/PD-L1+ | 0,5907 | 0,0125* |
| Survival since 1st recurrence | CD45+/CD86+ | 0,0049 | 0,9851 |
| Survival since 1st recurrence | CD45+/CD11b+ | -0,1422 | 0,5863 |
| Survival since 1st recurrence | CD45+/CD11b+/CD14+ | 0,0564 | 0,8298 |
| Survival since 1st recurrence | CD45+/CD11b+/CD14+/HLADR+ | 0,3057 | 0,2328 |
| Survival since 1st recurrence | CD45+/CD11b+/CD14+/PD-L1+ | 0,6324 | 0,0065* |
| Survival since 1st recurrence | CD45+/CD11b+/CD14+/CD86+ | -0,0637 | 0,8080 |
| Survival since 1st recurrence | Overall Survival | 0,8535 | <,0001* |
| Survival since vaccination | living cells | -0,0165 | 0,9481 |
| Survival since vaccination | HLADR+ | 0,1890 | 0,4527 |
| Survival since vaccination | PD-L1+ | 0,3523 | 0,1517 |
| Survival since vaccination | CD86+ | 0,0743 | 0,7694 |
| Survival since vaccination | CD45+ | 0,1834 | 0,4664 |
| Survival since vaccination | HLADR+/CD45+ | 0,1209 | 0,6328 |
| Survival since vaccination | CD45+/PD-L1+ | 0,3893 | 0,1103 |
| Survival since vaccination | CD45+/CD86+ | 0,1229 | 0,6272 |
| Survival since vaccination | CD45+/CD11b+ | -0,1208 | 0,6330 |
| Survival since vaccination | CD45+/CD11b+/CD14+ | 0,1208 | 0,6330 |
| Survival since vaccination | CD45+/CD11b+/CD14+/HLADR+ | 0,2260 | 0,3673 |
| Survival since vaccination | CD45+/CD11b+/CD14+/PD-L1+ | 0,4430 | 0,0656 |
| Survival since vaccination | CD45+/CD11b+/CD14+/CD86+ | 0,0279 | 0,9126 |
| Survival since vaccination | Overall Survival | 0,3487 | 0,1562 |
| Survival since vaccination | Survival since 1st recurrence | 0,7712 | 0,0005* |
| Survival since vaccination | Nr of vaccines | 0,6921 | 0,0015* |

**Supplementary Material Table S1. Spearman's non-parametric correlation.** The table reports the values of Spearman's coefficient (significant when >0.6), and the corresponding p-values, where significance levels are highlighted by the colors red ($p<0.05$) or orange ($p<0.01$ or $p<0.001$).
